# Evaluation of a Brief Personalised Intervention for Alcohol Consumption in College Students

**DOI:** 10.1371/journal.pone.0131229

**Published:** 2015-06-22

**Authors:** Natasha C. Clarke, Matt Field, Abigail K. Rose

**Affiliations:** 1 Department of Psychological Sciences, University of Liverpool, Liverpool, United Kingdom; 2 The UK Centre for Tobacco and Alcohol Studies (UKCTAS), Liverpool, United Kingdom; Penn State College of Medicine, UNITED STATES

## Abstract

In the current study we investigated the effect of a brief personalised feedback intervention (BPI), compared to an active control intervention, on outcome measures of (i) alcohol consumption (ii) frequency of binge drinking and (iii) readiness to change (RTC). A sample of 103 college students (mean age=23.85) who consumed alcohol regularly provided baseline measures of drinking behaviour and readiness to change before completing an alcohol-related quiz on the UK Department of Health’s Change4Life website (active control). The study was a between subjects design and half the participants were randomly allocated to the BPI group (N=52), who received 10 minutes personalised feedback on their drinking in addition to the alcohol-related quiz. At a two-week follow-up, participants (N=103) repeated the questionnaire battery, and attempted to recall the answers to the alcohol quiz. Results indicated that both groups significantly reduced their alcohol consumption and frequency of binge drinking but there were no significant group differences in either of these measures. We conclude that the provision of generalised information can be as efficient as a BPI for the reduction of alcohol consumption in students.

## Introduction

The UK Government recommends drinking should be kept within the lower risk category of 3–4 units for men and 2–3 units for women per day (UK unit = 8 g of alcohol) [1]. Excessive drinking, or binge drinking, is defined as consuming double this amount (≥8 units for men, ≥6 units for women) during one drinking occasion [2]. However, drinking over such guidelines is common, especially within college student populations. This issue is relevant to countries in several world regions (e.g. in Australasia, Europe, South American and North America [3]); in the US more than 80% of students drink alcohol and almost half report binge drinking in the last two weeks [4] (US binge definition: ≥5 drinks for men, ≥4 for women. Unit = 12 g alcohol [5]) and high levels of alcohol-related risk and harm (61%) are found in students within the UK [6]. With a constant increase in student numbers [7], college students represent a unique and high-risk group for alcohol-related harm and successful drink reduction strategies for this population would offer significant benefit for the individual and the wider society.

Policies regarding the dangerous use of alcohol often focus on harm-reduction methods. Current UK policy includes informing the public on alcohol guidelines so that individuals can make informed choices about healthier and responsible drinking, ultimately yielding a change in the broader drinking culture [8]. Information provision strategies have become widespread, but show disappointing results [9]. A review of mass media campaigns aimed at behavioural change concluded that in regards to alcohol consumption, they have had little success [10].

There are several possible explanations for the lack of effectiveness of information strategies. Firstly, people may not engage with or remember the information [11]. Jouriles and colleagues [12] found that recalling and writing down alcohol-related information led to a greater reduction in consumption after 2 weeks. Therefore increasing engagement with the information may mean it is remembered better and so is able to affect behaviour. Secondly, general harm-reduction strategies rarely fit the population as a whole, therefore policies may be more effective if personalised to the individual [13]. Brief personalised interventions (BPI) deliver short structured “brief advice” to encourage risky drinkers to lower their drinking levels [14], with content based on personalised information to increase an individuals’ awareness of their own behaviour [15]. Risky drinkers can be defined as ‘increasing risk’ drinkers (> 21 units for males and >14 units for females) or ‘higher risk’ drinkers (> 50 units of alcohol per week for males and > 35 units of alcohol per week for females) [16]. A web-based study involving Swedish students found that drinking assessment with personalised feedback resulted in fewer risky drinkers at 3 month follow-up compared with an assessment only and control group [17]. Similarly, a meta-analysis concluded that interventions containing personalised feedback are more effective than ‘assessment only’ control conditions in students [18]. However, compared to active controls (defined as brief-alternative alcohol-related interventions, general health interventions or interventions that provide only alcohol education) BPIs produced no additional benefit.

Research into BPIs in student populations has identified a number of elements which may help increase effectiveness: identifying consequences that are personally relevant; highlighting the practical costs of alcohol consumption; outlining risk reduction strategies; and including normative comparison [19]. All of these elements were contained in the BPI evaluated by the Screening and Intervention Programme for Sensible drinking (SIPS) trial [20]. This BPI was designed to provide practitioners with a structure to deliver brief advice to hazardous and harmful drinkers with an aim to capture risky drinking at an early stage, and provide advice or counselling to help reduce consumption [14]. SIPS assessed BPI effectiveness within a primary care setting but failed to find a difference between the BPI and control groups [20], perhaps indicating that different populations require different BPI elements.

Given that these BPI elements are not present in active controls, it is not clear why a difference in the effectiveness of these two approaches has not been found. It is possible that the personalised nature of the BPI elements encourages engagement with the intervention, and that it is this engagement which is key. The current study aimed to compare a BPI with an active control designed to encourage engagement (in the form of a quiz). Importantly, if both interventions are effective, this highlights that more cost effective harm reduction interventions (e.g., general information provision) may be effective in students, as long as engagement is successful.

One additional mechanism suggested to underlie drinking behaviour [21] is readiness to change (RTC), and some studies have found an increase in RTC scores following an intervention [22]. Therefore a further aim of BPIs in students may be to increase RTC [23]. In summary, the current study assessed the impact of a BPI, which includes the active elements previously identified as important in student populations, on alcohol consumption, frequency of binge drinking and readiness to change, compared with an active control (information-only) group in UK students. It was hypothesised that, compared to the active control, the BPI would result in (i) reduced fortnightly alcohol consumption and binge frequency and (ii) increased readiness to change.

## Method

### Participants

One hundred and three students (51 female; mean age 23.85 [SD ± 3.39]) were recruited from the University of Liverpool via advertisements, word of mouth and using the university’s online system. Previous brief intervention research has found small to medium effect sizes [15,24]. Power calculations using GPower [25] indicated that a sample size of 102 would detect a medium effect size (Cohen’s d = 0.5, with power (1—β) set at 0.80 and α = 05). Inclusion criteria were fluency in English and consumption of alcohol at least once per week. The study was approved by the University of Liverpool Research Ethics Committee. All participants provided written informed consent before taking part in the study, and they received £5 compensation for taking part.

### Measures

#### Alcohol Use Disorders Identification Test (AUDIT; [26])

The AUDIT is a screening tool designed to detect patterns of alcohol consumption that are hazardous to health [27]. When used in students it has been shown to have good internal consistency as a single factor (Cronbach’s alpha = 0.82; [28]).

#### Timeline Follow Back Questionnaire (TLFB;[29])

The TLFB is a self-report measure of retrospective alcohol consumption. In this version, participants recorded the number of alcohol units consumed over the previous two weeks. Outcome measures were average weekly alcohol consumption and the frequency of engaging in alcohol binges (binge defined as: ≥8 units p/drinking episode in men, ≥6 units p/drinking episode in women) [30].

#### Readiness to Change Contemplation Ruler (RTC; [31])

The contemplation ruler is a single item self-report measure, originally developed for smoking cessation [32]. Participants circled a response on a ruler ranging from 0 to 10, with 0 representing the statement “I never think about my drinking”, and 10 representing the statement “My drinking has changed. I now drink less than before”. The single-item ruler is highly correlated with the multiple-item RTC questionnaire (r = 0.77), and the ruler may be a better predictor of behavioural intentions than the RTCQ [31].

#### Active Control

Participants interacted with the alcohol section (‘Choose Less Booze’) of the Change4Life website (http://www.nhs.uk/Change4Life) to find the information needed to complete a quiz that comprised 10 questions related to alcohol guidelines, health risks and methods to reduce drinking. They were given a maximum of 15 minutes to complete the quiz, and the time taken to complete it was recorded.

#### Brief Personalised Intervention (BPI)

In addition to completing the alcohol quiz, as the Active Control group, participants received an alcohol BPI (administered by the researcher, single blind study) to encourage them to lower their drinking levels. The BPI was administered in a psychopharmacology laboratory on the University of Liverpool campus.

The BPI utilised a brief advice tool used in the SIPS alcohol screening and brief intervention (ASBI) research programme [14]. Participants received ten minutes personalised feedback on their alcohol use (based on TLFB and AUDIT data) in the form of face-to-face advice and a 2-page leaflet. The advice and leaflet assigned the participant to a drinking risk category (lower risk, increased risk, and high risk) based on government guidelines [33] and provided information about the health and social consequences of belonging to each of these categories. Participants were shown a sex specific graph, indicating that those in the increased and high-risk groups had drinking levels that exceeded the average for the population. They were also shown a list of benefits that would result from reduced drinking, advised about techniques that could help them to reduce their drinking, and provided with a personalised drinking reduction target.

### Procedure

A random number generator was used to randomly assign participants to either the BPI or active control group [34]. Participants provided informed consent before completing the initial questionnaire battery (RTC, TLFB, AUDIT). All participants then interacted with the alcohol section of the Change4Life website, and they were informed that they had a maximum of 15 minutes to find the information needed to answer the 10 alcohol-related questions in the quiz. Participants in the active control group were then discharged, whereas those in the BPI group received the personalised feedback intervention as described above.

#### Follow-up

All participants returned two weeks later. Participants completed the questionnaire battery (RTC, TLFB, AUDIT) before being given 15 minutes to complete the alcohol quiz given during the initial session (without access to the Change4Life website). A follow-up period of two weeks was used due to the interest in memory for the recall task; this was based on previous research with BPIs and the recall of information [12]. This was to assess level of engagement with the original task. Participants were then fully debriefed before being discharged.

## Results

Distribution of data was analysed and skewed variables were transformed to allow for parametric testing. There was one dropout in the control condition, who did not differ on baseline characteristics.

### Participant Characteristics (see Table 1)

MANOVA investigated whether participants across the two groups differed across independent measures (age, readiness to change ruler, AUDIT, weekly consumption, weekly binge). There were no significant differences at baseline between the groups (ps>0.05) and the groups did not differ by gender χ^2^(1, *N* = 103) = 0.79, *p* = 0.38. The sample was made up of 88.35% risky drinkers, identified by an AUDIT score of 8 or above [27]. Groups did not differ in percentage of risky drinkers χ^2^(1, *N* = 103) = 0.42, p = 0.52.

**Table 1:** Means (±SD) for participant characteristics by condition (N = 103).

	*Mean scores*(±*SD*)			*Statistics* (*One-way ANOVA*)	
Variable	*Intervention28 female 24 male*	*Control23 female28 male*	*Overall*	*F*	*p*
Age	23.63 (3.28)	24.08 (3.50)	23.85 (3.39)	0.32	0.57
RTC ruler	3.56 (2.12)	3.87 (2.62)	3.71 (2.37)	0.05	0.82
AUDIT	14.04 (5.54)	13.63 (5.36)	13.84 (5.43)	1.46	0.70
Fortnightly consumption (TLFB)	52.73 (32.03)	48.75 (39.53)	50.76 (35.83)	0.95	0.33
Weekly binge	1.53 (1.05)	1.51 (1.23)	1.52 (1.13)	0.20	0.65

TLFB = Time Line Follow Back; RTC ruler: Readiness to Change Ruler

* = significant at 0.05 level

### Changes in alcohol consumption

Changes in weekly alcohol unit consumption and binge frequency were investigated using mixed design 2x2 ANOVAs, with within-subject factors of time (2: baseline, follow-up) and between-subject factors of condition (2: BPI, active control).

**Fig 1:**
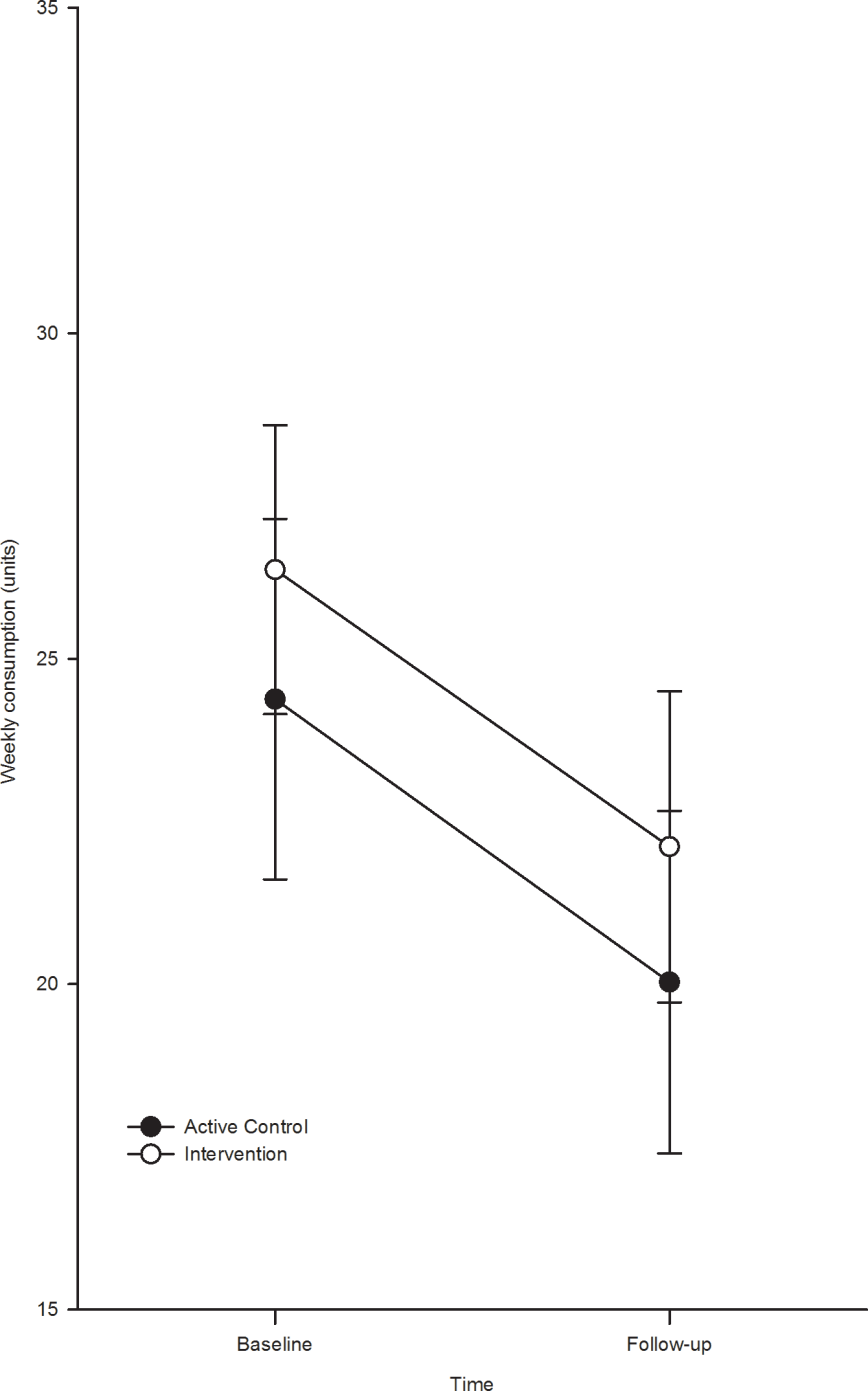
Weekly unit consumption at baseline and follow-up by condition.

The analysis of weekly unit consumption (Fig 1) revealed a significant main effect of time, F(1, 101) = 18.52, p = 0.001, ηp² = 0.16, with consumption decreasing significantly in both the BPI group, t(1, 51) = 3.24, p = 0.002 and the active control group t(1, 50) = 2.85, p = 0.006. The main effect of group, F(1, 101) = 0.48, p = 0.49 and the group x time interaction, F(1, 101) = 0.03, p = 0.86, were not statistically significant.

**Fig 2:**
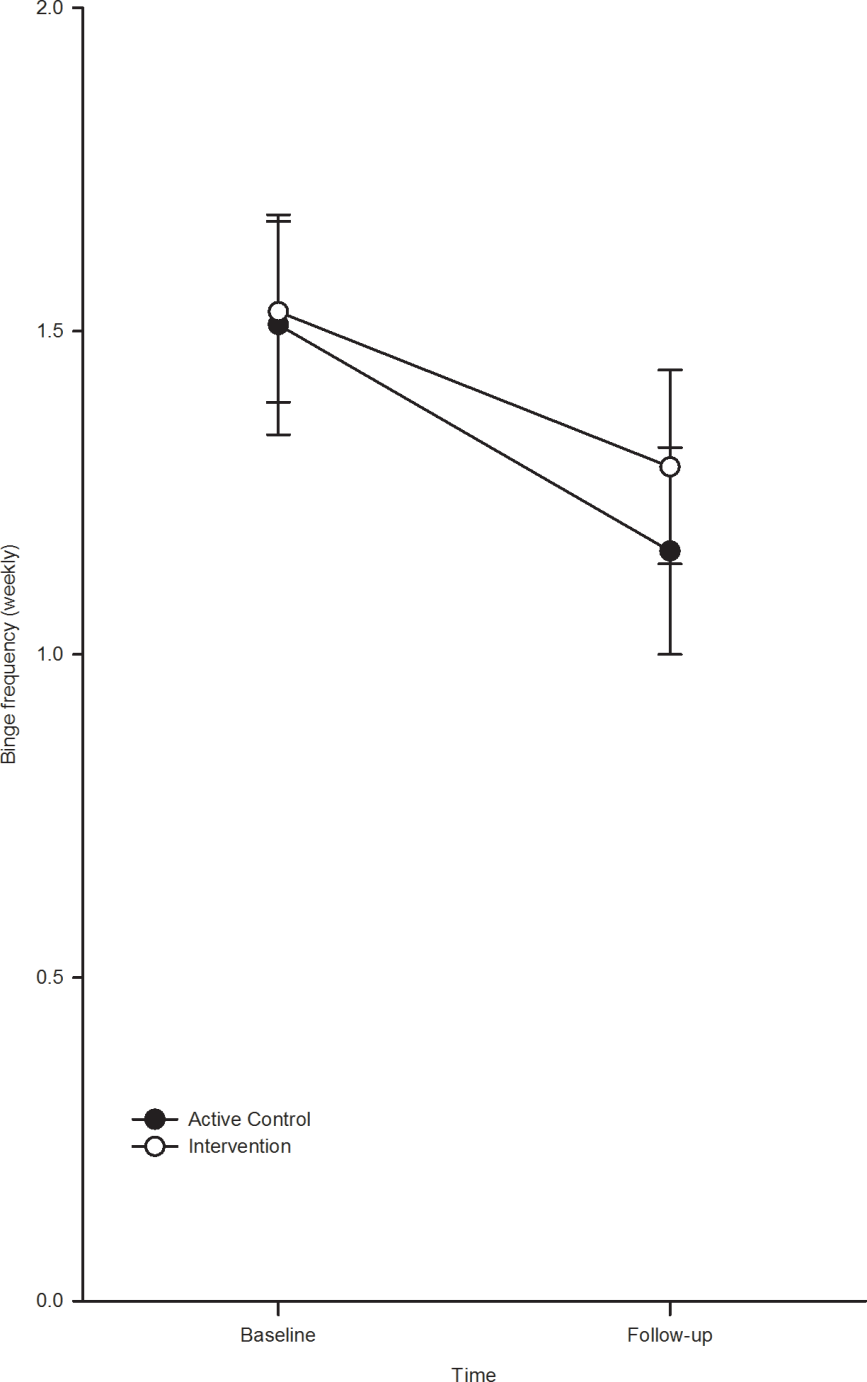
Weekly binge at baseline and follow-up by condition.

The analysis of binge frequency revealed an identical pattern (Fig 2). There was a significant main effect of time, F(1, 101) = 11.50, P = 0.001, ηp² = 0.10; binge frequency decreased in the BPI group, t(1, 51) = -2.41, p = 0.02 and the control group, t(1, 50) = 2.75, p = 0.01. The main effect of group, F(1, 101) = 0.38, p = 0.53 and the group x time interaction, F (1, 101) = 0.23, p = 0.63 were not statistically significant.

### Readiness to change (Fig 3)

Change in RTC was investigated using a mixed design 2x2 ANOVA, with within-subject factors of time (2: baseline, follow-up) and between-subject factors of condition (2: BPI, active control).

**Fig 3:**
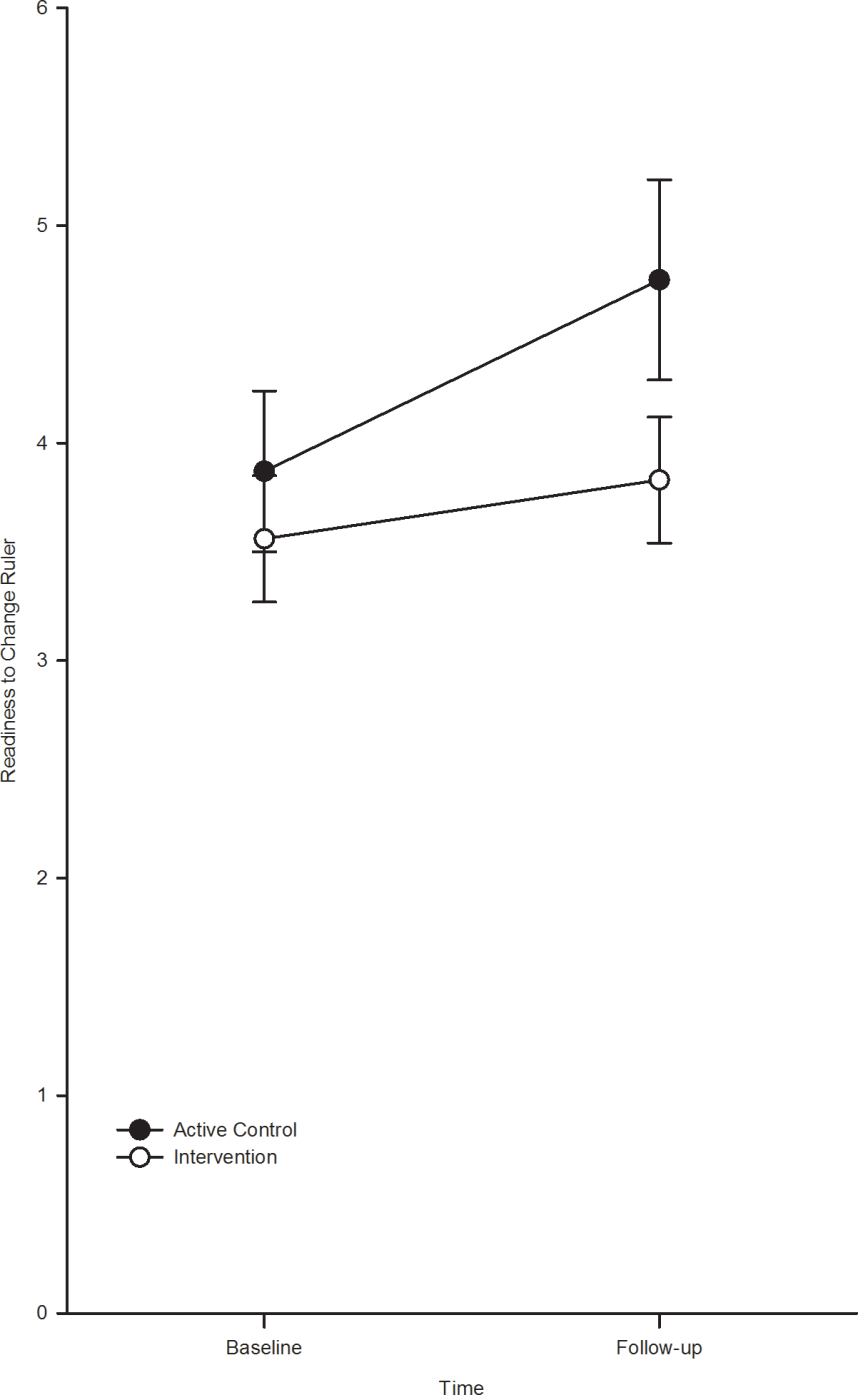
RTC ruler at baseline and follow-up by condition.

The analysis of RTC revealed a significant main effect of time, F (1, 101) = 5.7, p = 0.02, ηp² = 0.05, although post hoc t-tests indicated that the RTC increased between baseline and follow-up in the active control group, t(1, 51) = 2.05, p = 0.05, but not the BPI group, t(1, 51) = -0.97, p = 0.34. However, the main effect of group, F(1, 101) = 1.38, p = 0.24, and the group x time interaction, F(1, 101) = 1.06, p = 0.30, were not statistically significant.

### Experimental Task

One-Way ANOVA determined whether time taken to complete the alcohol quiz without access to the website [session 2] differed by group. There were no significant differences between groups for the time taken to complete the quiz, p = 0.91. There were no significant differences between groups for the recall of answers at follow-up, p = 0.36. Overall, participants recalled 50% of the information (ranging from 0–100%).

## Discussion

The present study compared the effect of a BPI with an active control on weekly alcohol consumption, binge frequency and readiness to change, in UK students. It was found that both groups significantly decreased their alcohol consumption and weekly binges to a similar extent.

The finding that both groups decreased their alcohol consumption significantly and by a similar amount suggests that the BPI offers no additional benefit to the active control [18]. This fits with previous research which has failed to find an effect of BPIs, compared with control, in reducing drinking within primary care settings [14]. It is possible that the SIPS’ BPI and control conditions may have contained similar active factors of behaviour change [35]; therefore the current findings may be due to specific components of the active control which included screening of quantity, frequency and consequences of the participants’ drinking [36]. Effects from assessment-only control groups is not a new phenomenon; small reductions on AUDIT scores and weekly alcohol consumption have been identified in control groups [37,38] and studies looking specifically at the effect of screening compared to non-screening have found significant effects on measures of hazardous drinking [39–41].

Most general information harm-reduction strategies simply provide individuals with various facts about alcohol and its harms. However, an important aspect of our study was that we encouraged engagement with the information-only component of the interventions by asking the participants to use the information to complete a quiz. Although research has suggested that information-only strategies are often ineffective [10], it is possible that they can be efficacious if engagement is maximised [12]. Information-only strategies are cheaper than BPIs [42] and so if we are to continue investing in information-only strategies, future research should identify ways to maximise engagement with the information. Our recall level at follow-up for both groups was approximately 50% which is significantly higher than that found by others (e.g, 10%) [12]. This suggests that quizzes may be one effective engagement strategy.

Another potential explanation for behaviour change in BPI and active control conditions is that both act upon the self-regulation of behaviour [38]. The process of reporting one’s own behaviour may result in reflecting on that behaviour which can trigger change. A possible mechanism is that by assessing our drinking behaviour, we become aware of risky practices, which initiates self-monitoring. Monitoring can allow us to recognise inconsistencies between current behaviour and a personal standard, which may impel us to change [43].

In terms of RTC, we hypothesised that the intervention would increase scores. However, there was no increase in the BPI group and there was no difference in RTC scores across groups. There have been mixed results with regards to interventions, alcohol use and motivation amongst students [44] with little success in regards to increases in RTC [45,46]. Evidence suggests that there is a non-linear relationship between alcohol use and RTC, as high levels of drinking are shown in those who show moderate levels of RTC, and low levels of drinking in those with high or low RTC [47]. A high variability on the RTC ruler both within and between individuals has also been found in previous research, with fluctuating alcohol consumption rates and scores on the ruler [48]. Taken together, research suggests RTC is a phenomenon difficult to measure and its relationship with alcohol is complex. It has been suggested an absence of consistent findings in this population of college students indicates the construct is of less importance for non-treatment seeking individuals [49].

There are a few points that need to be highlighted in terms of limitations. Firstly, most BPI research implements a screening method to identify risky drinkers before the intervention is administered [18], whereas the current study employed a universal approach. It could be argued that non-risky drinkers will show low RTC at baseline and therefore any effect of the intervention on RTC will be lost. However, over 88% of our sample were classed as risky drinkers so it is unlikely that RTC effects were missed. Additionally, one study found that abstinent students receiving personalised feedback were twice more likely to remain abstinent after one year than those in the control, indicating that in abstainers, feedback can function as a preventative measure [50]. Therefore, identifying which intervention components reduce risky drinking but also maintain non-risky drinking is important for future research. Although we note this was not an aim of this study and would require a longer follow-up period.

Secondly, the follow-up period of two weeks is a moderately short period to examine changes in drinking behaviour; therefore, firm conclusions regarding long-term effects cannot be drawn. Although many BPIs can be effective in the short term, the duration of this outcome is difficult to determine [19] with many studies demonstrating that drinking reduction effects diminish over time [51,52]. However, a recent meta-analysis of alcohol interventions for college students [18] found that effect size magnitude and intervention efficacy measures did not differ by assessment interval, apart from the frequency of heavy drinking, where it was found that when post-intervention assessments occurred one month or later they were more successful. Future research should include extended follow-up periods to determine how long any beneficial effects last and whether this differs across intervention type. If reductions do diminish over time, then future intervention development could involve ‘refresher’ interventions at regular intervals (e.g. via the internet or smartphone ‘apps’).

Importantly, as found in the majority of student studies [18], the current study demonstrates that in a UK student population a BPI offers no additional benefit to an active control at reducing alcohol consumption over a short time period. Given the risky drinking behaviour of this population, effective, evidence-based polices on alcohol harm-reduction strategies are needed. The active control, consisting of a drinking assessment and engagement with alcohol-related information, was as effective as a BPI in reducing alcohol-related behaviour. Active controls are arguably more cost effective than traditional BPIs which require more time and effort. If information-only interventions are suitable in this population, the next step will be to identify the best way to engage students in these strategies.

## Supporting Information

S1 Data Group averages by condition. (XLSX)

## References

[1] DoH (Department of Health). Sensible drinking: the report of an interdepartmental working group, London: Department of Health 1995.

[2] Office Cabinet. The Alcohol Harm Reduction Strategy for England. 3 2004 London: The Cabinet Office. 2004.

[3] Karam E, Kypri K, Salamoun M. Alcohol use among college students: An international perspective. Current Opinion in Psychiatry. 2007;20:213–221. 17415072 10.1097/YCO.0b013e3280fa836c

[4] National Institute on Alcohol Abuse and Alcoholism (NIAAA) College drinking. Fact sheet. 2013. Available: http://www.niaaa.nih.gov/alcohol-health/special-populations-co-occurring-disorders/college-drinking.

[5] National Institute on Alcohol Abuse and Alcoholism. National Institute of Alcohol Abuse and Alcoholism Council approves definition of binge drinking. NIAAA Newsletter 2004 Available: http://pubs.niaaa.nih.gov/publications/Newsletter/winter2004/Newsletter_Number3.htm.

[6] Heather N, Partington S, Partington E, Longstaff F, Allsop S, Jankowski M, et al Alcohol Use Disorders and Hazardous Drinking among Undergraduates at English Universities. 2011;46:270–277. 10.1093/alcalc/agr024 21450698

[7] Bolton P. Education: Historical Statistics. Social & General Statistics, Library, House of Commons; 2012.

[8] HM Government. The Government’s alcohol strategy. 2012. Available: https://www.gov.uk/government/uploads/system/uploads/attachment_data/file/224075/alcohol-strategy.pdf.

[9] Babor T, Holder H, Caetano R, Homel R, Casswell S, Livingston M, et al Alcohol: No Ordinary Commodity: Research and Public Policy Education and persuasion strategies. Oxford, Oxford University Press; 2010.

[10] Wakefield MA, Loken B, Hornik RC. Use of mass media campaigns to change health behaviour. The Lancet. 2010;376(9748):1261–71. 10.1016/S0140-6736(10)60809-4 20933263 PMC4248563

[11] McGuire LC. Remembering what the doctor said: organization and adults' memory for medical information. Exp Aging Res. 1996;22(4):403–28. 8968711 10.1080/03610739608254020

[12] Jouriles EN, Brown AS, Rosenfield D, McDonald R, Croft K, Leahy MM, et al Improving the effectiveness of computer-delivered personalized drinking feedback interventions for college students. Psychol Addict Behav. 2010;24(4):592–9. 10.1037/a0020830 21198222

[13] Marlatt GA, Witkiewitz K. Update on harm-reduction policy and intervention research. Annu Rev Clin Psychol. 2010;6:591–606. 10.1146/annurev.clinpsy.121208.131438 20192791

[14] Kaner E, Bland M, Cassidy P, Coulton S, Dale V, Deluca P, et al Effectiveness of screening and brief alcohol intervention in primary care (SIPS trial): pragmatic cluster randomised controlled trial. Bmj. 2013;346. 10.1136/bmj.e8501 PMC3541471 23303891

[15] Riper H, van Straten A, Keuken M, Smit F, Schippers G, Cuijpers P. Curbing problem drinking with personalized-feedback interventions: a meta-analysis. American journal of preventive medicine. 2009;36(3):247–55. 10.1016/j.amepre.2008.10.016 19215850

[16] Public Health England. Who are increasing and higher risk drinkers? PHE Alcohol Learning Resources; 2010. Available: http://www.alcohollearningcentre.org.uk/Topics/Browse/SocialMarketing/toolkit2010/introduction/IncreasingAndHigherRisk/.

[17] McCambridge J, Bendtsen M, Karlsson N, White IR, Nilsen P, Bendtsen P. Alcohol assessment and feedback by email for university students: main findings from a randomised controlled trial. The British journal of psychiatry: the journal of mental science. 2013;203(5):334–40. 10.1192/bjp.bp.113.128660 24072758 PMC3814613

[18] Scott-Sheldon LA, Carey KB, Elliott JC, Garey L, Carey MP. Efficacy of alcohol interventions for first-year college students: a meta-analytic review of randomized controlled trials. J Consult Clin Psychol. 2014;82(2):177–88. 10.1037/a0035192 24447002 PMC3987817

[19] Miller MB, Leffingwell T, Claborn K, Meier E, Walters S, Neighbors C. Personalized feedback interventions for college alcohol misuse: an update of Walters & Neighbors (2005). Psychol Addict Behav. 2013;27(4):909–20. 10.1037/a0031174 23276309 PMC4948182

[20] Drummond C, Deluca P, Coulton S, Bland M, Cassidy P, Crawford M, et al The effectiveness of alcohol screening and brief intervention in emergency departments: a multicentre pragmatic cluster randomized controlled trial. Plos One. 2014;9(6):e99463 10.1371/journal.pone.0099463 24963731 PMC4070907

[21] Rollnick S, Heather N, Gold R, Hall W. Development of a short 'readiness to change' questionnaire for use in brief, opportunistic interventions among excessive drinkers. British journal of addiction. 1992;87(5):743–54. 1591525 10.1111/j.1360-0443.1992.tb02720.x

[22] Ostafin BD, Palfai TP. When wanting to change is not enough: automatic appetitive processes moderate the effects of a brief alcohol intervention in hazardous-drinking college students. Addiction science & clinical practice. 2012;7:25. 23217219 10.1186/1940-0640-7-25 PMC3685546

[23] Larimer ME, Cronce JM, Lee CM, Kilmer JR. Brief interventions in college settings. Alcohol Research and Health. 2004;28:94–104. 19006997 PMC6601644

[24] Moyer A, Finney JW, Swearingen CE, Vergun P. Brief interventions for alcohol problems: a meta-analytic review of controlled investigations in treatment-seeking and non-treatment-seeking populations. Addiction. 2002; 97(3):279–292. 11964101 10.1046/j.1360-0443.2002.00018.x

[25] Faul F, Erdfelder E. GPOWER: A priori-, post hoc-, and compromise power analyses for MS-DOS [Computer Program]. Bonn, Germany: Bonn University; 1992

[26] Saunders JB, Aasland OG, Babor TF, de la Fuente JR, Grant M. Development of the Alcohol Use Disorders Identification Test (AUDIT): WHO Collaborative Project on Early Detection of Persons with Harmful Alcohol Consumption—II. Addiction. 1993;88(6):791–804. 8329970 10.1111/j.1360-0443.1993.tb02093.x

[27] Babor TF, Higgins-biddle JC, Saunders JB, Monteiro M. AUDIT: The Alcohol Use Disorders Identification Test Guidelines for Use in Primary Care Department of Mental Health and Substance Dependence. World Health Organisation, London; 2001

[28] Shields AL, Guttmannova K, Caruso JC. An examination of the factor structure of the Alcohol Use Disorders Identification Test in two high-risk samples. Substance use & misuse. 2004;39(7):1161–82. 15387208 10.1081/ja-120038034

[29] Sobell L, Sobell M. Timeline Follow-Back In Litten R. & Allen J. (Eds.), Measuring Alcohol Consumption: Humana Press; 1992 pp. 41–72.

[30] NICE. Alcohol-use disorders: preventing harmful drinking. 2010. Available https://www.nice.org.uk/guidance/ph24/chapter/1-recommendations

[31] LaBrie JW, Quinlan T, Schiffman JE, Earleywine ME. Performance of alcohol and safer sex change rulers compared with readiness to change questionnaires. Psychology of Addictive Behaviors, 2005;19:112–115. 15783287 10.1037/0893-164X.19.1.112

[32] Biener L, Abrams DB. The Contemplation Ladder: validation of a measure of readiness to consider smoking cessation. Health psychology: official journal of the Division of Health Psychology, American Psychological Association. 1991;10(5):360–5. 10.1037//0278-6133.10.5.360 1935872

[33] Edwards G. Sensible drinking. Bmj. 1996;312(7022):1 8555842 10.1136/bmj.312.7022.1 PMC2349701

[34] Roberts C, Torgerson D. Randomisation methods in controlled trials BMJ (Clin Res) 1998;317:1301 9804722 10.1136/bmj.317.7168.1301 PMC1114206

[35] Abraham C, Michie S. A taxonomy of behavior change techniques used in interventions. Health psychology: official journal of the Division of Health Psychology, American Psychological Association. 2008;27(3):379–87. 10.1037/0278-6133.27.3.379 18624603

[36] Dimeff L, Baer J, Kivlahan D, Marlatt G. Brief alcohol screening and intervention for college students (BASICS), New York: Guilford Press; 1999.

[37] Jenkins RJ, McAlaney J, McCambridge J. Change over time in alcohol consumption in control groups in brief intervention studies: systematic review and meta-regression study. Drug Alcohol Depend. 2009;100(1–2):107–14. 10.1016/j.drugalcdep.2008.10.017 19041196

[38] McCambridge J, Kypri K. Can simply answering research questions change behaviour? Systematic review and meta analyses of brief alcohol intervention trials. Plos One. 2011;6(10):e23748 10.1371/journal.pone.0023748 21998626 PMC3187747

[39] Carey KB, Carey MP, Maisto SA, Henson JM. Brief motivational interventions for heavy college drinkers: A randomized controlled trial. J Consult Clin Psychol. 2006;74(5):943–54. 17032098 10.1037/0022-006X.74.5.943 PMC2442891

[40] McCambridge J, Day M. Randomized controlled trial of the effects of completing the Alcohol Use Disorders Identification Test questionnaire on self-reported hazardous drinking. Addiction. 2008;103(2):241–8. 10.1111/j.1360-0443.2007.02080.x 18199302

[41] Walters ST, Vader AM, Harris TR, Jouriles EN. Reactivity to Alcohol Assessment Measures: An Experimental Test. Addiction. 2009;104(8):1305–10. 10.1111/j.1360-0443.2009.02632.x 19624323 PMC2724752

[42] World Health Organisation. Alcohol. Fact sheet; 2014. Available: http://www.who.int/mediacentre/factsheets/fs349/en/.

[43] Moos RH. Context and mechanisms of reactivity to assessment and treatment. Addiction. 2008;103(2):249–50. 10.1111/j.1360-0443.2007.02123.x 18199303

[44] Shealy AE, Murphy JG, Borsari B, Correia CJ. Predictors of motivation to change alcohol use among referred college students. Addict Behav. 2007;32(10):2358–64. 17398012 10.1016/j.addbeh.2007.02.003 PMC2726646

[45] Fromme K, Corbin W. Prevention of heavy drinking and associated negative consequences among mandated and voluntary college students. J Consult Clin Psychol. 2004;72(6):1038–49. 15612850 10.1037/0022-006X.72.6.1038

[46] Schaus JF, Sole ML, McCoy TP, Mullett N, O'Brien MC. Alcohol Screening and Brief Intervention in a College Student Health Center: A Randomized Controlled Trial. Journal of Studies on Alcohol and Drugs Supplement. 2009;16:131–41. 19538921 10.15288/jsads.2009.s16.131 PMC2701092

[47] Cadigan JM, Martens MP, Arterberry BJ, Smith AE, Murphy JG. Examining a curvilinear model of readiness to change and alcohol consumption. Addict Res Theory. 2013;21(6):507–15. 24696671 10.3109/16066359.2012.754884 PMC3970817

[48] Kaysen DL, Lee CM, LaBrie JW, Tollison SJ. Readiness to Change Drinking Behavior in Female College Students. Journal of Studies on Alcohol and Drugs Supplement. 2009;16:106–14. 19538918 10.15288/jsads.2009.s16.106 PMC2701096

[49] Collins SE, Logan DE, Neighbors C. Which came first: the readiness or the change? Longitudinal relationships between readiness to change and drinking among college drinkers. Addiction. 2010;105(11):1899–909. 10.1111/j.1360-0443.2010.03064.x 20854333 PMC3934960

[50] Larimer ME, Lee CM, Kilmer JR, Fabiano PM, Stark CB, Geisner IM, et al Personalized Mailed Feedback for College Drinking Prevention: A Randomized Clinical Trial. Journal of Consulting and Clinical Psychology. 2007;75:285–93. 17469886 10.1037/0022-006X.75.2.285 PMC3271786

[51] Carey KB, Scott-Sheldon LA, Carey MP, DeMartini KS. Individual-level interventions to reduce college student drinking: a meta-analytic review. Addict Behav. 2007;32(11):2469–94. 17590277 10.1016/j.addbeh.2007.05.004 PMC2144910

[52] Carey KB, Carey MP, Maisto SA, Henson JM. Computer Versus In-Person Intervention for Students Violating Campus Alcohol Policy. Journal of consulting and clinical psychology. 2009;77(1):74–87. 10.1037/a0014281 19170455 PMC2657221

